# Clozapine Normalizes a Glutamatergic Transmission Abnormality Induced by an Impaired NMDA Receptor in the Thalamocortical Pathway via the Activation of a Group III Metabotropic Glutamate Receptor

**DOI:** 10.3390/biom9060234

**Published:** 2019-06-17

**Authors:** Kouji Fukuyama, Ryo Kato, Masahiko Murata, Takashi Shiroyama, Motohiro Okada

**Affiliations:** 1 Department of Neuropsychiatry, Division of Neuroscience, Graduate School of Medicine, Mie University, Tsu 514-8507, Japan; mk_psy_isui@hotmail.com (K.F.); ryo-kato@clin.medic.mie-u.ac.jp (R.K.); takashi@clin.medic.mie-u.ac.jp (T.S.); 2 National Hospital Organization Sakakibara Hospital, 777 Sakakibara, Tsu, Mie 514-1292, Japan; muyuhton@gmail.com

**Keywords:** clozapine, schizophrenia, NMDA/glutamate receptor, metabotropic glutamate receptor, thalamocortical pathway

## Abstract

Pharmacological mechanisms of gold-standard antipsychotics against treatment-refractory schizophrenia, such as clozapine (CLZ), remain unclear. We aimed to explore the mechanisms of CLZ by investigating the effects of MK801 and CLZ on tripartite synaptic transmission in the thalamocortical glutamatergic pathway using multi-probe microdialysis and primary cultured astrocytes. l-glutamate release in the medial prefrontal cortex (mPFC) was unaffected by local MK801 administration into mPFC but was enhanced in the mediodorsal thalamic nucleus (MDTN) and reticular thalamic nucleus (RTN) via GABAergic disinhibition in the RTN–MDTN pathway. The local administration of therapeutically relevant concentrations of CLZ into mPFC and MDTN increased and did not affect mPFC l-glutamate release. The local administration of the therapeutically relevant concentration of CLZ into mPFC reduced MK801-induced mPFC l-glutamate release via presynaptic group III metabotropic glutamate receptor (III-mGluR) activation. However, toxic concentrations of CLZ activated l-glutamate release associated with hemichannels. This study demonstrated that RTN is a candidate generator region in which impaired *N*-methyl-d-aspartate (NMDA)/glutamate receptors likely produce thalamocortical hyperglutamatergic transmission. Additionally, we identified several mechanisms of CLZ relating to its superiority in treatment-resistant schizophrenia and its severe adverse effects: (1) the prevention of thalamocortical hyperglutamatergic transmission via activation of mPFC presynaptic III-mGluR and (2) activation of astroglial l-glutamate release associated with hemichannels. These actions may contribute to the unique clinical profile of CLZ.

## 1. Introduction

Schizophrenia is considered to be a heterogeneous disorder that is unlikely to be caused by a single etiological factor, but rather by a complex network of interacting pathogenic influences [1]. It has been established that dysfunctions of both dopaminergic and glutamatergic transmission play important roles in the pathophysiology of schizophrenia, with various antipsychotics improving dopaminergic hyperfunction in the mesolimbic pathway and hypofunction in the mesocortical pathway [2,3]. Impairment of the *N*-methyl-d-aspartate (NMDA)/glutamate receptor (NMDA-R) also contributes to the pathophysiology of schizophrenia [2,3,4,5]. Indeed, several clinical reports have demonstrated that NMDA-R antagonists (e.g., phencyclidine and ketamine) can generate schizophrenia-like positive and negative symptoms in healthy volunteers [2,6,7] and exacerbate psychosis in patients with schizophrenia [8]. Moreover, NMDA-R antagonist-induced psychosis models exhibit features of schizophrenia, such as negative symptoms and cognitive deficits, more closely than the amphetamine/dopamine psychosis models [9]. Based on clinical evidence, dysfunctional glutamatergic transmission associated with NMDA-R seems to produce a schizophrenia-like state.

Unexpectedly, the systemic administration of the non-competitive NMDA-R antagonist, MK801, increased glutamate release in the medial prefrontal cortex (mPFC) [10,11,12,13,14] and enhanced glutamatergic neuronal activity in the mPFC [15,16]. In contrast to systemic administration, local MK801 administration into the mPFC by reverse dialysis did not affect extracellular glutamate levels in the mPFC [17,18,19]. These discrepant effects between systemic and local administrations of MK801 into the mPFC on l-glutamate release in the mPFC indicate that the generator region for systemic MK801-induced l-glutamate release in the mPFC is outside the mPFC. Indeed, local MK801 administration into the mediodorsal thalamic nucleus (MDTN) was shown to increase extracellular glutamate levels in the mPFC [13,14]. The half maximal inhibitory concentration (IC_50_) of MK801 for NMDA-R ranges from 5 to 50 nM [20]. However, in our recent study, local administration of more than 25 μM of MK801 (estimated to be 3.6 μM in the brain tissue concentration) into the MDTN increased l-glutamate release in the mPFC, whereas 5 μM (estimated to be 3.6 μM in the brain tissue concentration) had no effect [13,14]. The discrepancy between the IC_50_ values and the effective MK801 concentration suggests that the thalamocortical glutamatergic pathway (i.e., MDTN–mPFC) contributes to systemic MK801-induced l-glutamate release in the mPFC, whereas the generator of MK801-induced l-glutamate release is probably outside the MDTN. In another recent study, we showed that glutamatergic neurons in the MDTN project glutamatergic terminals to the frontal cortex and receive inhibitory projections that affect gamma-aminobutyric acid (GABA) from the reticular thalamic nucleus (RTN) using multiprobe microdialysis [21]. Various thalamic nuclei, including the MDTN, receive GABAergic inhibitory input from the RTN; the RTN receives projections from various cortices, limbic system and basal ganglia [22,23]. Thalamic GABAergic inhibition is considered to be the integrator of motor and sensory processing, perception, and cognition [23,24].

Clozapine (CLZ) is considered the most effective antipsychotic but also the most toxic [25]. Indeed, despite causing myocarditis, cardiomyopathy, agranulocytosis, and convulsions [26], CLZ remains the gold-standard antipsychotic for treatment-refractory schizophrenia and is the only approved medication licensed for this indication [25,27]. An effect on NMDA-R function may be involved in the antipsychotic efficacy of CLZ [28] based on evidence from a double-blind, placebo-controlled clinical study in which CLZ significantly blunted a ketamine-induced increase in positive symptoms [29]. The astroglial releases of l-glutamate and D-serine induced by CLZ are one of the major mechanisms of agonistic action of CLZ against NMDA-R [28]. Thalamocortical glutamatergic transmission (i.e., MDTN–mPFC) is regulated by the cystine/glutamate antiporter (system xc-: Sxc) and by group II and III metabotropic glutamate receptors (II-mGluR and III-mGluR, respectively) [13,14]. Indeed, atypical antipsychotics (such as aripiprazole) and cognitive enhancers (such as memantine and amantadine) have been shown to inhibit MK801-induced l-glutamate release in the mPFC via modulation of II-mGluR and Sxc in the mPFC and MDTN, respectively [13,14,30], while CLZ has been shown to inhibit phencyclidine-induced hyperlocomotion in wild-type and II-mGluR knockout mice [31]. Recently, a preclinical behavior study demonstrated that CLZ prevented antipsychotic-related behavioral effects of a II-mGluR agonist [32]. Thus, although activation of Sxc or III-mGluR are possibly involved in the antipsychotic efficacy of CLZ, the effects of CLZ on Sxc and III-mGluR are yet to be clarified.

A pre-clinical study demonstrated that CLZ protected neuronal damage by inhibiting several redox and proinflammatory responses [33,34]. In contrast, CLZ elevates the risk of convulsions compared with other atypical antipsychotics [25,35]. These clinical and preclinical data suggest that there is a double-edge sword concerning the mechanisms of action of CLZ. The dysfunction of glial gap-junctions contributes to attenuated information processing and cognitive impairment, which are major symptoms of schizophrenia [1]. Gap-junctions and hemichannels are integral components of neuronal excitability, synaptic plasticity, tripartite synaptic transmission, and homeostasis maintenance in the central nervous system. However, pathological conditions, including ischemia and excessive depolarization, generate persistent gap-junction and hemichannel opening, which leads to the disruption of several homeostasis systems [36,37]. In particular, inhibitors of gap-junctions and hemichannels can prevent the onset of epileptic seizures [37,38,39]. In the context of our previous research, we primarily aimed to explore the detailed mechanisms of MK801-induced glutamate release in the mPFC to determine the regulatory mechanisms of glutamatergic transmission in the RTN–MDTN–mPFC pathway. Overall, our aim was to further our understanding of the pathophysiology of schizophrenia and the mechanisms of action of CLZ, focusing on thalamocortical tripartite synaptic transmission by multiprobe microdialysis with primary cultured astrocytes.

## 2. Materials and Methods

### 2.1. Chemical Agents

Clozapine was purchased from Sigma (St. Louis, MO, USA). The non-competitive NMDA-R antagonist, MK801 [40], was obtained from Wako Chemicals (Osaka, Japan). The II-mGluR antagonist (LY341495), the III-mGluR antagonist ((RS)-α-cyclopropyl-4-phosphonophenyl glycine (CPPG)) [41], and the Sxc inhibitor ((S)-4-carboxyphenylglycine (CPG)) [30,42] were purchased from Tocris Bioscience (Bristol, UK). The hemichannel and gap-junction blocker, carbenoxolone (CBX) [43], was obtained from Cosmo Bio (Tokyo, Japan).

LY341495 and CBX were initially dissolved in 10 mM with dimethyl sulfoxide, then diluted to 1 mM with modified Ringer’s solution (MRS) or artificial cerebrospinal fluid (ACSF) [28,44]. CPPG and CPG were dissolved in MRS or ACSF. CLZ and MK801 were dissolved in MRS, ACSF, or Dulbecco’s modified Eagle’s medium containing 10% fetal calf serum (fDMEM) containing less than 0.1% acetic acid.

### 2.2. Preparation of the Microdialysis System

All animal care and experimental procedures described in this report complied with the Ethical Guidelines established by the Institutional Animal Care and Use Committee at Mie University, Japan (No. 24–35–R2) and are reported in accordance with the Animal Research: Reporting of In Vivo Experiments (ARRIVE) guidelines [45]. A total of 144 rats were used. Rats were anesthetized with 1.8% isoflurane and placed in a stereotaxic frame. Concentric direct insertion type dialysis probes were implanted in the mPFC (A = +3.2 mm, L = +0.8 mm, V = −5.2 mm, relative to the bregma; 0.22 mm diameter, 3 mm exposed membrane; Eicom, Kyoto, Japan), MDTN (A = −3.0 mm, L = +0.9 mm, V = −6.2 mm, relative to the bregma at a lateral angle of 30°; 0.22 mm diameter, 2 mm exposed membrane; Eicom), and RTN (A = −1.4 mm, L = +1.2 mm, V = −7.2 mm, relative to the bregma at a lateral angle of 30°) [46].

Perfusion experiments commenced 18 h after recovery from anesthesia [28,44] using a constant rate of 1 μL/min with MRS (145 Na^+^, 2.7 K^+^, 1.2 Ca^2+^, 1.0 Mg^2+^, and 154.4 Cl^−^, buffered to a pH of 7.4 with a 2 mM phosphate buffer and 1.1 mM Tris buffer) [28,44]. Extracellular levels of l-glutamate and GABA were measured at 8 h after starting the perfusion. When the coefficients of variation for l-glutamate reached less than 5% over 60 min (stabilization), control data were obtained over another period of 60 min (pre-treatment period). This was followed by intraperitoneal (i.p.) administration of MK801 or a local perfusion of each agent. To determine the effects of each agent, the perfusion medium was then switched to MRS containing the target agent. Each dialysate was injected into the ultra-high-performance liquid chromatography (UHPLC) apparatus. The location of the dialysis probes was verified at the end of each experiment using 200 μm thick brain tissue slices (Vibratome 1000, Technical Products International INC, St. Louis, MO, USA)2.3. Preparation of Primary Astrocyte Culture

Pregnant Sprague-Dawley rats (SLC, Sizuoka, Japan), which were housed individually in cages, kept in air-conditioned rooms (with a temperature of 22 ± 2 °C) set to a 12 h light/dark cycle, and given free access to food and water. Cultured astrocytes were prepared from cortical astrocyte cultures of neonatal Sprague-Dawley rats (*N* = 18) sacrificed by decapitation at 0–24 h of age. The cerebral hemispheres were removed under a dissecting microscope. Tissue was chopped into fine pieces using scissors, then triturated briefly with a micropipette. A suspension was filtered using a 70 µm nylon mesh (BD, Franklin Lakes, NJ, USA) and centrifuged. Pellets were then re-suspended in 10 mL fDMEM, which was repeated three times. After culture for 14 days (DIV14), contaminating cells were removed by shaking in a standard incubator for 16 h at 200 rpm. On DIV21, astrocytes were removed from flasks by trypsinization and seeded directly onto a translucent PET membrane (1.0 μm) with 24-well plates (BD) at a density of 1 × 105 cells/cm^2^ for experiments [13,14,44,47]. From DIV21 to DIV28, the culture medium was changed twice a week, and various agents were added for chronic administrations (seven days). On DIV28, cultured astrocytes were washed out using ACSF, and this was repeated three times. The ACSF comprised 130 mM NaCl, 5.4 mM KCl, 1.8 mM CaCl_2_, 1 mM MgCl_2_, and 5.5 mM glucose. It was buffered to a pH of 7.3 with a 20 mM HEPES buffer [30,48]. The remaining adherent cells contained 95% glial fibrillary acidic protein (GFAP)-positive and A2B5-negative cells detected using immunohistochemical staining [49]. After the wash-out, astrocytes were incubated in ACSF (100 μL translucent polyethylene terephthalate (PET) membrane) at 35 °C for 60 min in a CO_2_ incubator (pre-treatment incubation). They were then incubated in ACSF containing the agents (60 min) for acute administration and collection of the ACSF for analysis. Each 100 μL of collected ACSF was filtered by Vivaspin 500-3K (Sartorius, Goerringen, Germany) and freeze-dried for storage at −80 °C until needed for analyses.

### 2.3. Determination of Levels of l-Glutamate and GABA

Levels of l-glutamate and GABA were determined by the fluorescence resonance energy transfer method [50,51] using UHPLC (xLC3185PU, Jasco) with a fluorescence detector (xLC3120FP, Jasco, Tokyo, Japan) after dual derivatization with isobutyryl-l-cysteine and o-phthalaldehyde [28,44,47]. Derivative reagent solutions were prepared by dissolving isobutyryl-l-cysteine (2 mg) and o-phthalaldehyde (1 mg) in 0.1 mL ethanol, followed by the addition of 0.9 mL sodium borate buffer (0.2 M, pH of 9.0). Automated pre-column derivatives were obtained by drawing up a 5 μL aliquot of the standard or blank solution and 5 μL of the derivative reagent and holding them in vials for 5 min before injection. The derivatized samples (5 μL) were injected using an autosampler (xLC3059AS, Jasco). An analytical column (YMC Triat C18, particle size of 1.8 μm, 50 × 2.1 mm, YMC, Kyoto, Japan) was maintained at 45 °C and a flow rate of 500 μL/min. Linear gradient elution was performed for over 10 min in mobile phases A (0.05 M citrate buffer, pH of 5.0) and B (0.05 M citrate buffer containing 30% acetonitrile and 30% methanol, pH of 3.5). The excitation/emission wavelengths of the fluorescence detector were set at 280 and 455 nm, respectively [50,51].

### 2.4. Determination of Diffusion Rates of CLZ and MK801

To measure concentrations of CLZ and MK801 accurately in brain tissue perfused into the RTN, MDTN, and mPFC, in vivo microdialysis probe diffusion was determined according to a reverse dialysis procedure [52,53]. Because solute diffusion occurs in both directions across dialysis membranes, loss of solute from the perfusate occurs at the same rate as recovery of the solute into the perfusate. During analyses, the temperature was maintained at 37 °C with a perfusion warmer. The probe was set in the warmer chamber, and the MRS containing CLZ or MK801 was perfused into the probe for 180 min.

Levels of CLZ and MK801 were determined by UHPLC (PU-4185, Jasco) with a mass spectrometer (Acquity SQ detector, Waters, Milford, MA, USA). We then injected 5 μL aliquots of filtered samples using the autosampler and determined the concentrations of CLZ and MK801 using the UHPLC instrument equipped with a Triart C18 column (particle 1.8 μm, 50 × 2.1 mm, YMC) column at 40 °C with a mobile phase set to 500 µL/min (Acquity UPLC, Waters). A linear gradient elution program was performed over 10 min with mobile phases A (5 mM ammonium acetate buffer, pH of 9.0) and B (acetonitrile). Nitrogen flows for desolvation and in the cone were set at 750 and 50 L h^−1^, respectively, with the desolvation temperature set to 450 °C. The cone voltages for CLZ (*m*/*z* = 327.8) and MK801 (*m*/*z* = 222.2) were 20 and 25 V, respectively.

The diffusion rates for CLZ and MK801 were calculated as previously reported [14]. The losses of CLZ and MK801 from the dialysis probes (internal to external) were 9.6 ± 0.5% and 14.4 ± 1.9%, respectively. The estimated concentrations of CLZ in extracellular spaces during perfusion with 10, 30, and 100 μM CLZ were 0.96, 2.8, and 9.6 μM, respectively. The estimated concentrations of MK801 in extracellular spaces during perfusion with 1, 10, and 50 μM MK801 were 0.1, 1.4, and 7.2 μM, respectively.

### 2.5. Statistical Analysis

**Figure 1:**
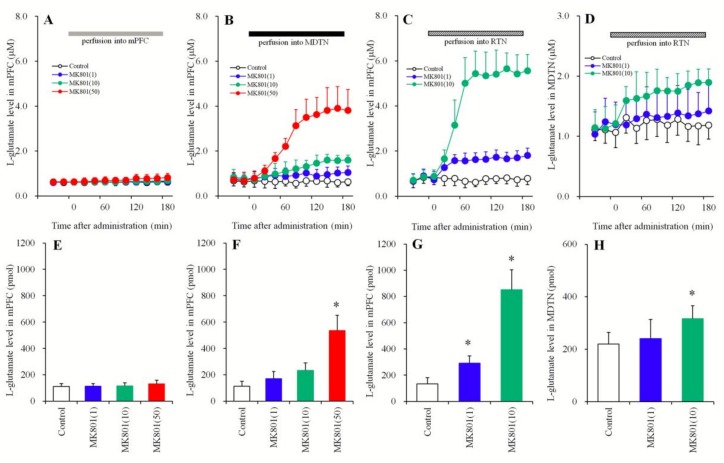
Concentration-dependent effects of local administration (perfusion) of MK801 into the medial prefrontal cortex (mPFC) (**A**,**E**), the mediodorsal thalamic nucleus (MDTN) (**B**,**F**), and the reticular thalamic nucleus (RTN) (**C**,**G**) on the extracellular l-glutamate level in the mPFC and perfusion of MK801 into the RTN on the extracellular l-glutamate level in the MDTN (**D**,**H**). The perfusion medium commenced with MRS. After confirming the stabilization of extracellular l-glutamate levels, the perfusates in the mPFC (A: gray column), MDTN (B: black column), and RTN (CD: stripped column) were switched to MRS containing MK801 (1, 10, or 50 μM) for 180 min. Ordinates: mean ± standard deviation (SD) (*n* = 6) of extracellular l-glutamate levels (μM). Abscissas: time after administration of MK801 perfusion (min). Area under curve (AUC) values of l-glutamate levels (pmol) during perfusion with MK801 (from 20 to 180 min) based on (**A**–**D**) were indicated in (**E**–**H**), respectively. * *p* < 0.05. This is relative to the control (opened bar) using an LME with Tukey’s post hoc test.

**Figure 2:**
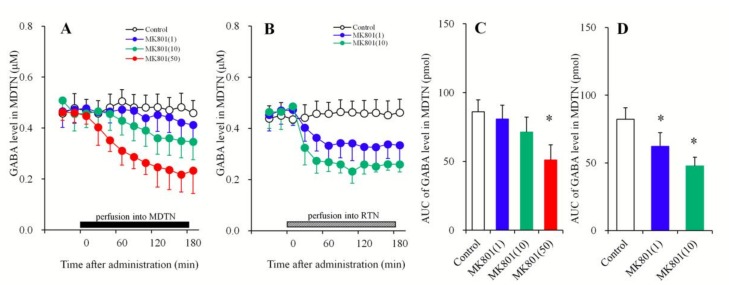
Concentration-dependent effects of local administration (perfusion) of MK801 into the MDTN (**A**,**C**) and RTN (**B**,**D**) on extracellular GABA levels in the MDTN. The perfusion medium commenced with MRS. After the confirming the stabilization of extracellular GABA levels in the MDTN, the perfusate in the MDTN (A: black column) and RTN (B: stripped column) was switched to MRS containing MK801 (1, 10, or 50 μM) for 180 min. Ordinates: mean ± SD (*n* = 6) of extracellular GABA levels (μM). Abscissa: time after administration of MK801 perfusion (min). AUC values of GABA levels (pmol) during perfusion with MK801 (from 20 to 180 min) based on (**A**,**B**) were indicated in (**C**,**D**), respectively. * *p* < 0.05. This is relative to the control (opened bar) using LME with Tukey’s post hoc test.

**Figure 3:**
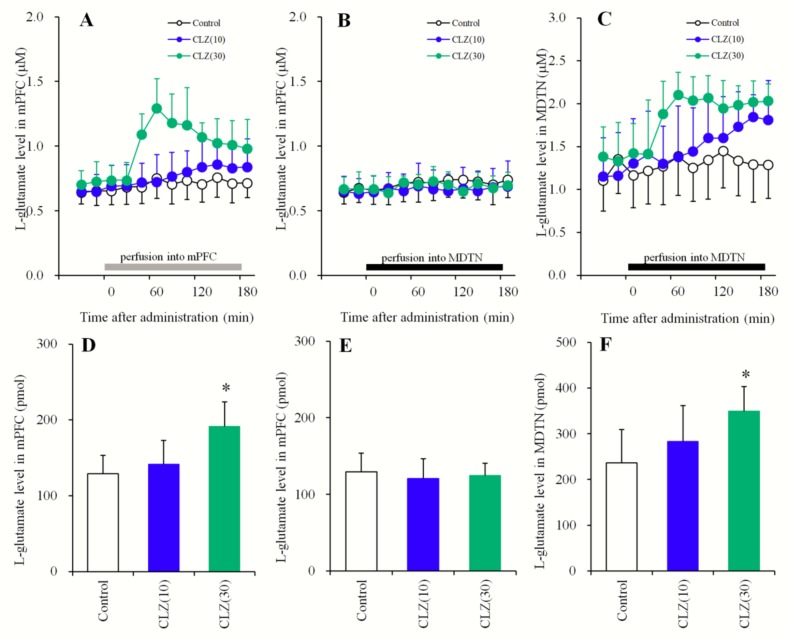
Concentration-dependent effects of the local administration (perfusion) of CLZ into the mPFC (**A**,**D**) and MDTN (**B**,**E**) on extracellular l-glutamate levels in the mPFC, and the perfusion into the MDTN on levels in the MDTN (**C**,**F**). The perfusion medium commenced with MRS. After confirming the stabilization of extracellular l-glutamate levels, the perfusate in the mPFC (A: gray column) or MDTN (BC: black columns) was switched to MRS containing CLZ (10 or 30 μM) for 180 min. Ordinates: mean ± SD (*n* = 6) of extracellular l-glutamate levels (μM). Abscissas: time after administration of CLZ perfusion (min). AUC values of l-glutamate levels (pmol) during perfusion with CLZ (from 20 to 180 min) based on (**A**–**C**) were indicated in (**D**–**F**), respectively. * *p* < 0.05. This is relative to the control (opened bar) using LME with Tukey’s post hoc test.

**Figure 4:**
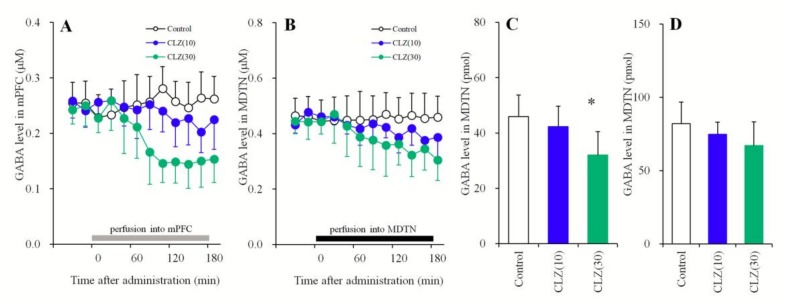
Concentration-dependent effects of the local administration (perfusion) of CLZ into the mPFC on extracellular GABA levels in the mPFC (**A**,**C**) and the perfusion of CLZ into the MDTN on levels in the MDTN (**B**,**D**). The perfusion medium commenced with MRS. After confirming the stabilization of extracellular GABA levels, the perfusate in the mPFC (A: gray column) or MDTN (B: black column) was switched to MRS containing CLZ (10 or 30 μM) for 180 min. Ordinates: mean ± SD (*n* = 6) of extracellular GABA levels (μM). Abscissas: time after administration of CLZ perfusion (min). AUC values of GABA levels (pmol) during perfusion with CLZ (from 20 to 180 min) based on (**A**,**B**) were indicated in (**C**,**D**), respectively. * *p* < 0.05. This is relative to the control (opened bar) using LME with Tukey’s post hoc test.

**Figure 5:**
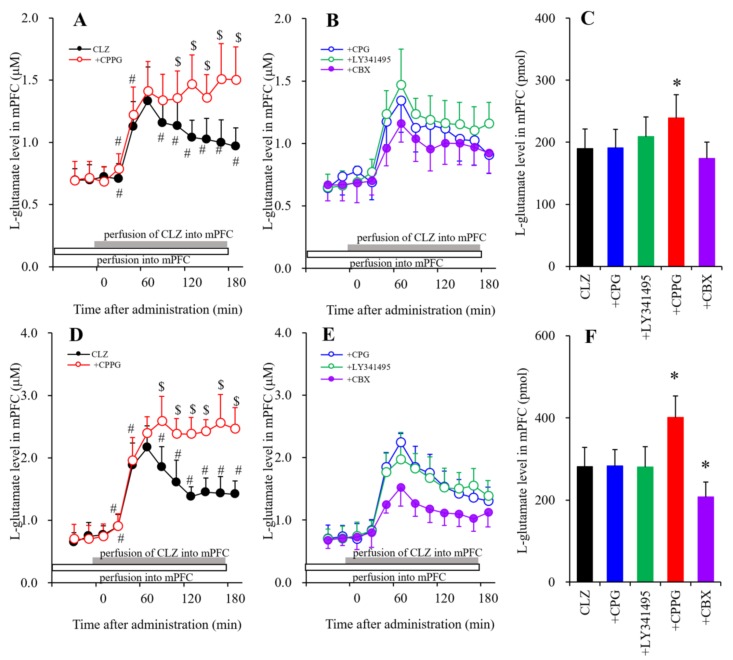
Interaction of perfusions with CLZ (**A**–**C**): 30 and (**D**–**F**): 100 μM) and CPG (1 μM), LY341495 (1 μM), CPPG (100 μM), and CBX (100 μM) into the mPFC on extracellular l-glutamate levels in the mPFC. Perfusion medium in the mPFC commenced with MRS containing CPG, LY341495, CPPG, or CBX. After confirming the stabilization of extracellular l-glutamate levels, the perfusate in the mPFC was switched to the same perfusate containing CLZ (30 or 100 μM) for 180 min. Ordinates: mean ± SD (*n* = 6) of extracellular l-glutamate levels (μM). Abscissas: time after administration of CLZ perfusion (min). AUC values of l-glutamate levels (pmol) during perfusion with CLZ (from 20 to 180 min) based on (**A**,**B**) (30 μM CLZ) and (**D**,**E**) (100 μM CLZ) were indicated in Figure 5C,F, respectively. * *p* < 0.05. This is relative to the control (CLZ: black bar) using LME with Tukey’s post hoc test. In (**A**,**D**), ^#^ *p* < 0.05. This is relative to the l-glutamate at 60 min (^$^ *p* < 0.05). This is relative to CLZ using LME with Tukey’s post hoc test.

**Figure 6:**
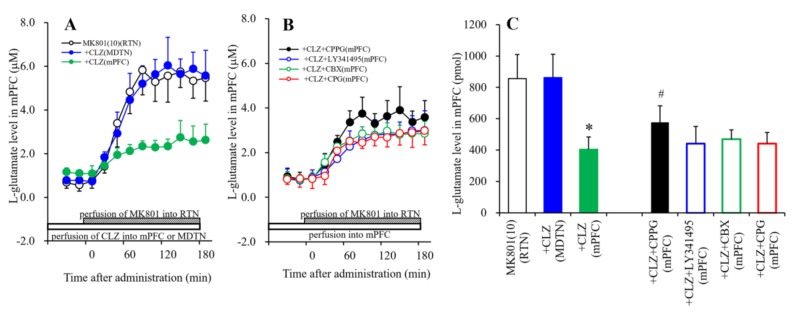
Effects of perfusion with therapeutically relevant concentration of CLZ (30 μM) into the mPFC and MDTN on mPFC l-glutamate release induced by perfusion with MK801 (10 μM) into the RTN (**A**,**B**). Perfusion medium in the mPFC or MDTN commenced with MRS containing CLZ. After confirming the stabilization of extracellular l-glutamate levels, the perfusate in the RTN was switched from MRS to MRS containing MK801 for 180 min. Interaction between perfusion with CLZ (30 μM) and CPG (1 μM), LY341495 (1 μM), CPPG (100 μM), or CBX (100 μM) into the mPFC and its impact on mPFC l-glutamate release induced by perfusion with MK801 (10 μM) into the RTN (**B**,**C**). Perfusion medium in the mPFC commenced with MRS containing CLZ with CPG, LY341495, CPPG, or CBX. After confirming the stabilization of extracellular l-glutamate levels, the perfusate in the RTN was switched from MRS to MRS containing MK801 for 180 min. Ordinates: mean ± SD (*n* = 6) of extracellular l-glutamate level (μM). Abscissas: time after administration of MK801 perfusion (min). AUC values of l-glutamate level (pmol) during perfusion with MK801 (from 20 to 180 min) based on (**A**,**B**) were indicated in (**C**). * *p* < 0.05. This is relative to the control (MK801: opened bar) and ^#^ *p* < 0.05. This is relative to MK801 + CLZ (mPFC) (green bar) using LME with Tukey’s post hoc test.

**Figure 7:**
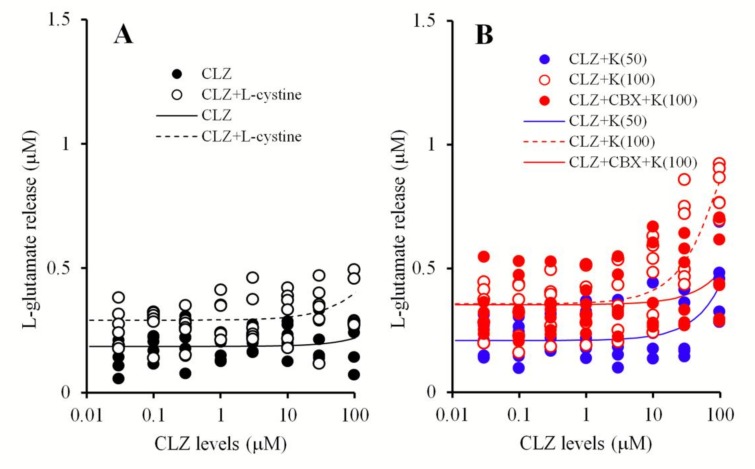
Concentration-dependent effects of CLZ (0.03–100 μM) on basal ganglia and 100 mM cystine-induced releases of l-glutamate from primary cultured astrocytes (**A**). Interaction between CLZ and CBX on 50 and 100 mM K^+^-evoked l-glutamate releases from primary cultured astrocytes (**B**). Ordinates: mean ± SD (*n* = 6) of extracellular l-glutamate levels (μM). Abscissas: CLZ concentration (μM). Concentration-dependent effects of CLZ on astroglial l-glutamate release was statistically analyzed using logistic regression analysis.

All experiments were designed with equal-sized groups (*n* = 6) that were predetermined based on our previous studies [13,14,21,48]. All values are expressed as mean ± standard deviation (SD), and a *p*-value less than 0.05 was considered statistically significant. Analyses were performed in IBM SPSS for Windows, Version 25 (IBM Corp., Armonk, NY, USA) and BellCurve for Excel ver. 3.00 (Social Survey Research Information Co., Ltd., Tokyo, Japan). The concentration-dependent effects of the local administration of MK801 (1, 10, and 50 μM) and CLZ (30, 100, and 300 μM) on extracellular levels of l-glutamate and GABA were compared using a linear mixed effects model (LME) (IBM SPSS), followed by Tukey’s post hoc test (BellCurve) when the *F*-value of the concentration factor was significant (Figure 1, Figure 2, Figure 3 and Figure 4). The effects of MK801, CLZ, LY341495, (RS)-α-cyclopropyl-4-phosphonophenyl glycine (CPPG), (S)-4-carboxyphenylglycine (CPG), and carbenoxolone (CBX) on an extracellular l-glutamate level were compared using LME, followed by Tukey’s post hoc test when the *F*-value of the drug factor was significant (Figure 5 and Figure 6). Particularly, the effect of the local administration of CPPG into the mPFC on the biphasic kinetics of l-glutamate release induced by CLZ was analyzed by LME followed by Tukey’s post hoc test when the *F*-value is the reciprocal factor of CPPG × time (Figure 5A,D). The concentration-dependent effects of CLZ on basal and on cystine- and K^+^-evoked astroglial l-glutamate release from primary cultured astrocytes were analyzed using logistic regression analysis (BellCurve) (Figure 7). The data and statistical analysis comply with the recommendations on experimental design and analysis in pharmacology [54].

## 3. Results

### 3.1. Microdialysis Study

#### 3.1.1. Concentration-Dependent Effects of the Local Administration of MK801 into the mPFC, MDTN, and RTN on Extracellular l-Glutamate Levels in the mPFC and the MDTN

In order to clarify the responsible region of l-glutamate release in the mPFC induced by systemic MK801 (a noncompetitive NMDA-R inhibitor), the concentration-dependent effects of local MK801 administration into the mPFC, MDTN, and RTN on l-glutamate release in the mPFC were determined using multiprobe microdialysis. Perfusion with MK801 (1, 10, and 50 μM) into the mPFC did not affect the extracellular l-glutamate level in the mPFC (Figure 1A,E), but perfusion into the MDTN concentration-dependently increased the extracellular l-glutamate level in the mPFC [F_MK801_ (3,20) = 34.9 (*p* < 0.01), F_Time_ (9,239) = 88.4 (*p* < 0.01), F_MK801 *Time_ (27,239) = 45.0 (*p* < 0.01)] (Figure 1B,F). Perfusion with MK801 at 50 μM into the MDTN increased the extracellular l-glutamate level in the mPFC, whereas perfusion with lower concentrations of MK801 (1 and 10 μM) did not (Figure 1B,F). Similarly, perfusion with MK801 (1 and 10 μM) into the RTN concentration-dependently increased the extracellular L-glutamate level in the mPFC [F_MK801_(2,15) = 83.0 (*p* < 0.01), F_Time_ (9,135) = 88.4 (*p* < 0.01), F_MK801*Time_ (18,135) = 45.0 (*p* < 0.01)] (Figure 1C,G) and MDTN [F_MK801_ (2,15) = 4.0 (*p* < 0.05), F_Time_(9,135) = 11.8 (*p* < 0.01), F_MK801*Time_ (18,135) = 4.4 (*p* < 0.01)] (Figure 1D,H). Notably, perfusion with MK801 at 10 μM into the RTN increased extracellular l-glutamate levels in both the mPFC and MDTN (Figure 1C,D,G,H), whereas 1 μM increased levels in the mPFC but not in the MDTN (Figure 1C,D,G,H).

#### 3.1.2. Concentration-Dependent Effects of the Local Administration of MK801 into the MDTN and the RTN on Extracellular GABA Levels in the MDTN

In order to clarify the mechanisms of mPFC l-glutamate release induced by local MK801 administration into the MDTN and RTN, the concentration-dependent effects of local MK801 administration (1, 10, and 50 μM) into the MDTN and RTN on GABA release in the MDTN were determined using multiprobe microdialysis. 

Perfusion with MK801 (1, 10, and 50 μM) into the MDTN concentration-dependently decreased extracellular GABA levels in the MDTN [F_MK801_ (3,20) = 11.5 (*p* < 0.01), F_Time_ (9,239) = 77.4 *(p <* 0.01), F_MK801*Time_ (27,239) = 20.9 (*p* < 0.01)] (Figure 2A,C). Perfusion with MK801 (50 μM) into the MDTN decreased extracellular GABA levels in the MDTN, whereas perfusion with MK801 (1 and 10 μM) into the MDTN did not affect this (Figure 2A,C). Perfusion with MK801 (1 and 10 μM) into the RTN concentration-dependently decreased extracellular GABA level in the MDTN [F_MK801_(2,15) = 19.1 (*p* < 0.01), F_Time_ (9,135) = 52.5 (*p* < 0.01), F_MK801*Time_ (18,135) = 21.9 (*p* < 0.01)] (Figure 2B,D). Contrary to the perfusion of MK801 into the MDTN, both 1 and 10 μM MK801 significantly decreased extracellular GABA levels in the MDTN (Figure 2B,D).

#### 3.1.3. Concentration-Dependent Effects of the Local Administration of CLZ into the mPFC and the MDTN on Extracellular l-Glutamate Levels in the mPFC and MDTN

Survival analysis has suggested that effective relapse prevention requires the maintenance of patients at CLZ serum concentrations above 200 μg/L (0.6 μM) [55]; however, exceeding 1300 μg/L (4 μM) can significantly increase the risk of adverse effects such as seizures [56]. Considering the diffusion rate through the dialysis probe, perfusion concentrations of 10 or 30 μM CLZ were considered therapeutically relevant (estimated to be 0.96 and 2.8 μM in the brain tissue concentration, respectively), whereas perfusion with 100 μM CLZ (9.6 μM in brain tissue) was considered toxic [55,56].

In order to study the concentration-dependent effects of CLZ on thalamocortical glutamatergic transmission, the effects of the local administration of therapeutically relevant concentrations of CLZ (10 and 30 μM) into the mPFC and MDTN on l-glutamate release in the mPFC and MDTN were determined using multiprobe microdialysis. Perfusion with CLZ (10 and 30 μM) into the mPFC concentration-dependently increased extracellular l-glutamate levels in the mPFC [F_CLZ_ (2,15) = 6.5 (*p* < 0.01), F_Time_ (9,135) = 26.9 (*p* < 0.01), F_CLZ*Time_ (18,135) = 13.1 (*p* < 0.01)] (Figure 3A,D). Perfusion with CLZ (30 μM) into the mPFC increased extracellular l-glutamate levels in the mPFC, whereas CLZ (10 μM) did not affect (Figure 3A,D). Perfusion with CLZ (10 and 30 μM) into the MDTN did not affect extracellular l-glutamate levels in the mPFC (Figure 3B,E), but it concentration-dependently increased levels in the MDTN [F_CLZ_(2,15) = 3.4 (*p* < 0.05), F_Time_ (9,135) = 22.2 (*p* < 0.01), F_CLZ*Time_ (18,135) = 7.2 (*p* < 0.01)] (Figure 3C,F). Perfusion with CLZ (30 μM) into the MDTN increased extracellular l-glutamate levels in the MDTN, whereas CLZ (10 μM) did not affect those levels (Figure 3C,F).

#### 3.1.4. Concentration-Dependent Effects of the Local Administration of CLZ into the mPFC and MDTN on Extracellular GABA Levels in the mPFC and MDTN

In order to clarify the mechanism of CLZ-induced l-glutamate release, the effects of the local administration of therapeutically relevant concentrations of CLZ (10 and 30 μM) into the mPFC and MDTN on GABA release in the respective areas were determined using microdialysis. Perfusion with CLZ (10 and 30 μM) into the mPFC concentration-dependently decreased extracellular GABA levels in the mPFC [F_CLZ_(2,15) = 4.4 (*p* < 0.05), F_Time_(9,135) = 22.3 (*p* < 0.01), F_CLZ*Time_(18,135) = 20.9 (*p* < 0.01)] (Figure 4A,C). Perfusion with CLZ (30 μM) into the mPFC decreased extracellular GABA levels in the mPFC, whereas perfusion with CLZ (10 μM) into the mPFC did not affect those levels (Figure 4A,C). Contrary to the mPFC, perfusion with CLZ (10 and 30 μM) into the MDTN did not affect extracellular GABA levels in the MDTN (Figure 4B,D).

#### 3.1.5. The Interaction of the Perfusion with CLZ and the Modulator of mGluRs and Hemichannels into the mPFC on Extracellular l-Glutamate Levels in the mPFC

To study the mechanisms of CLZ-induced l-glutamate release in the mPFC, the perfusion medium in the mPFC commenced with MRS containing or not containing (control) an Sxc inhibitor, CPG (1 μM) [30], II-mGluR antagonist, LY341495 (1 μM), III-mGluR antagonist, CPPG (100 μM) or hemichannel inhibitor, and CBX (100 μM). After stabilization, the perfusion medium was switched to the same MRS containing therapeutically relevant (30 μM, Figure 5A,B) or toxic (100 μM, Figure 5D,E) doses of CLZ for 180 min. 

Perfusion with CLZ (30 μM) into the mPFC increased extracellular l-glutamate levels in the mPFC (Figure 5A,C). The stimulatory effect of tharapeutic-relevant concentrations of CLZ on extracellular l-glutamate release in the mPFC was enhanced by III-mGluR inhibition (perfusion with 100 μM CPPG) [F_CPPG_(1,10) = 5.2 (*p* < 0.05), F_Time_(9,90) = 110.5 (*p* < 0.01), F_CPPG*Time_(9,90) = 18.5 (*p* < 0.01)] (Figure 5A,C), but not by 10 μM CBX (hemichannel inhibitor), 1 μM LY341495 (II-mGluR antagonist), or 1 μM CPG into the mPFC (Figure 5B,C). Notably, 30 μM of induced CLZ increases l-glutamate levels in two phases: an initial rise phase (from 20 to 60 min) and a late attenuation phase (from 80 to 180 min). CPPG did not affect the initial rise, only inhibiting the late attenuation. 

Perfusion with toxic concentrations of CLZ (100 μM) into the mPFC increased extracellular l-glutamate levels in the mPFC (Figure 5D,F). As with 30 μM CLZ, 100 μM CLZ induced an increase in l-glutamate levels with an initial rise (from 20 to 60 min) and a late attenuation (from 80 to 180 min). The stimulatory effect of perfusion with CLZ (100 μM) into the mPFC on extracellular l-glutamate release in the mPFC was enhanced and reduced by perfusion with both CPPG (100 μM) [F_CPPG_ (1,10) = 14.8 (*p* < 0.05), F_Time_ (9,90) = 230.2 (*p* < 0.01), F_CPPG*Time_ (9,90) = 44.6 (*p* < 0.01)] (Figure 5D,F) and CBX (100 μM) [F_CBX_(1,10) = 8.1 (*p* < 0.05), F_Time_(9,90) = 135.9 (*p* < 0.01), F_CBX*Time_ (9,90) = 13.9 (*p* < 0.01)] into the mPFC, respectively (Figure 5B,D). Again, CPPG did not affect the initial rise, but did inhibit late attenuation. However, CBX inhibited the initial rise, but not the late attenuation (Figure 5E,F). 

#### 3.1.6. Interaction between MK801 and CLZ and its Impact on Extracellular l-Glutamate Levels in the mPFC

To study the effects of CLZ in the mPFC and MDTN on MK801-induced L-glutamate release in the mPFC by perfusion with MK801 into the RTN, perfusion medium in the mPFC and MDTN commenced with MRS containing a therapeutically relevant concentration of CLZ (30 μM). After stabilization, the perfusion medium in the RTN was switched from MRS to MRS containing MK801 (10 μM) (Figure 6A). Perfusion with MK801 (10 μM) into the RTN increased extracellular l-glutamate levels in the mPFC (Figure 6A). This MK801-induced l-glutamate release was inhibited by perfusion with CLZ (30 μM) into the mPFC, but not by perfusion with CLZ (30 μM) into the MDTN [F_CLZ_ (1,10) = 36.6 (*p* < 0.05), F_Time_ (9,90) = 134.2 (*p* < 0.01), F_CPPG*Time_ (9,90) = 46.0 (*p* < 0.01)] (Figure 6A,C). 

To clarify the inhibitory effects of CLZ in the mPFC on MK801-induced l-glutamate release in the mPFC by perfusion with MK801 into the RTN, perfusion medium in the mPFC commenced with MRS containing CLZ (30 μM) with CPG (1 μM), LY341495 (1 μM), CPPG (100 μM), or CBX (100 μM). After stabilization, the perfusion medium in the RTN was switched from MRS to MRS containing MK801 (10 μM) (Figure 6B). The inhibitory effect of perfusion with CLZ (30 μM) into the mPFC was ameliorated by perfusion with CPPG (100 μM), but was not affected by LY341495 (1 μM), CPG (1 μM), or CBX (100 μM) [F_Agents_ (2,15) = 16.9 (*p* < 0.01), F_Time_ (9,135) = 274.9 (*p* < 0.01), F_Agents*Time_ (18,135) = 25.0 (*p* < 0.01)] (Figure 6B,C).

### 3.2. Primary Cultured Astrocyte Study

#### Effects of Concentration-Dependent Effects of CLZ on Hemichannel and Sxc Activities of Primary Cultured Astrocytes

CLZ (at concentrations of 0.03 to 100 μM) did not affect astroglial L-glutamate release from primary cultured astrocytes (Figure 7A). Similarly, it did not affect astroglial l-glutamate release induced by 100 μM l-cystine (Sxc activity) [14,30] (Figure 7A). Astroglial l-glutamate release concentration-dependently increased extracellular K^+^ levels (Figure 7B). CLZ weakly enhanced K^+^-evoked astroglial l-glutamate release at concentrations above 30 μM, but the EC_50_ value was not detectable (Figure 7B). Notably, 100 mM K^+^-evoked astroglial l-glutamate release was inhibited by 100 μM CBX (Figure 7B).

## 4. Discussion

### 4.1. Candidate Mechanisms of MK801-Induced l-Glutamate Release in the mPFC

**Figure 8:**
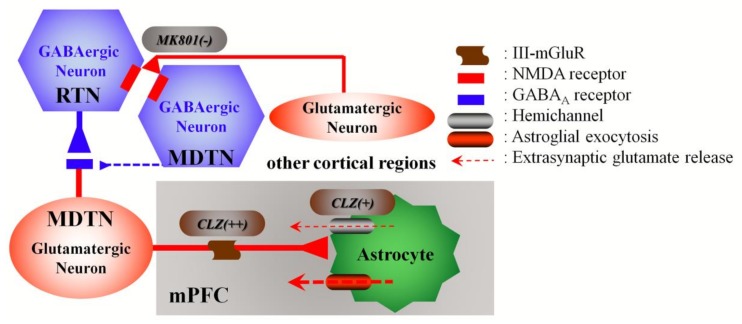
Our proposed hypothesis for the extended neural circuitry involved in thalamocortical (RTN–MDTN–mPFC) glutamatergic transmission. Glutamatergic neurons in the MDTN (red circle), which receive GABAergic terminals from RTN [22,23] and other MDTN regions, project to mPFC [21]. Inhibitory GABAergic regulation of MDTN glutamatergic neurons from the RTN is predominant rather than regulation of those from the MDTN. MK801 inhibits the actions of tonically active NMDA-R (red squares) on GABAergic neurons (blue hexagon) in the RTN and the MDTN. Inhibition of NMDA-R in GABAergic neurons leads to disinhibition of MDTN glutamatergic neurons. The GABAergic disinhibition activates MDTN glutamatergic neuronal activity, resulting in an increase in glutamate release in the mPFC. CLZ increased l-glutamate release in the mPFC via astroglial and pre-synaptic regulation mechanisms. Therapeutically relevant concentrations of CLZ activates the function of pre-synaptic III-mGluRs (brown wave) and extra-synaptic astroglial exocytosis (red ellipse) [28] in the mPFC region. Toxic concentrations of CLZ activates hemichannel (gray ellipse) activity. CLZ prevents hyperactivated MDTN–mPFC glutamatergic transmission via activation of inhibitory pre-synaptic III-mGluRs.

Several studies have demonstrated that the systemic administration of the non-competitive NMDA-R antagonists, phencyclidine [10], ketamine [11], and MK801 [12] increase glutamate release in the mPFC. However, it has also been shown that local administration of MK801 [12,17,18,19,28,49] and ketamine [11] into the mPFC had no effect on glutamate release in the mPFC. In contrast, local administration of higher MK801 concentrations (25 and 50 μM) into the MDTN have been shown to increase l-glutamate levels in the mPFC, unlike conventional concentrations (1 and 10 μM) which had no effect [13,14]. The RTN projects GABAergic terminals to various thalamic nuclei, including the MDTN [22,23] (Figure 8). The present study demonstrated that the local administration of MK801 (1 and 10 μM) into the RTN decreased MDTN GABA release and increased l-glutamate release in the MDTN and mPFC. Therefore, inhibition of NMDA-R in the RTN leads to GABAergic disinhibition in the NDTN (RTN–MDTN), resulting in enhanced thalamocortical glutamatergic transmission (MDTN–mPFC). In other words, we discovered that the RTN is a candidate generator region of l-glutamate release induced by systemic MK801 administration in the mPFC (Figure 8). 

### 4.2. CLZ Regulates Thalamocortical Glutamatergic Transmission

The thalamocortical glutamatergic pathway receives inhibitory regulation through III-mGluR in the mPFC and through II-mGluR in both the MDTN and mPFC via counter-exported l-glutamate from astroglial Sxc [13,14]. Cognitive enhancers, such as aripiprazole, memantine, and amantadine inhibited MK801-induced L-glutamate release in the mPFC and basal ganglia by activating II-mGluR and Sxc in the mPFC, MDTN, and globus pallidus, respectively [13,14,30]. In the present study, MK801-induced L-glutamate release was inhibited by local CLZ administration into the mPFC but not the MDTN. Furthermore, the inhibitory effects of CLZ in the mPFC were prevented by the III-mGluR antagonist, CPPG. Given that III-mGluR is localized in the presynaptic active zone [57], hyperactivation of glutamatergic transmission in the MDTN–mPFC induced by GABAergic disinhibition in the RTN–MDTN can be improved by presynaptic III-mGluR activation in the mPFC. Although both aripiprazole and CLZ prevent NMDA-R-induced behavioral dysfunction, we conclude that their mechanisms differ in terms of the location on which they act upon. Based on previous and present studies, we speculate that the target region of CLZ is the mPFC rather than the thalamus. This is supported by the behavior study showing that CLZ inhibits phencyclidine-induced hyperlocomotion independently of II-mGluR, (i.e., it affected both wild-type and II-mGluR knockout mice) [31]. Furthermore, a recent epigenetic study demonstrated that CLZ attenuated the performance of II-mGluR agonists [32]. 

Despite inhibition thalamocortical hyperglutamatergic transmission, systemic administration of CLZ has been reported to increase l-glutamate and decrease GABA in the mPFC [13,14,28,58]. CLZ-induced l-glutamate release in the mPFC is thought to include astroglial l-glutamate release [28]. The present study added that the local administration of CLZ into the mPFC also increased l-glutamate and decreased GABA releases in the mPFC. Moreover, local CLZ administration into the MDTN increased L-glutamate and decreased GABA releases in the MDTN without affecting l-glutamate release in the mPFC. These results suggest that the mPFC is probably the generator region of CLZ-induced L-glutamate release in the mPFC. Therefore, systemic administration of either MK801 or CLZ increased L-glutamate release in the mPFC, but the mechanisms of these two agents differ. 

The inhibitory effects of CLZ on MK801-induced thalamocortical hyperglutamatergic transmission are modulated by the activation of inhibitory presynaptic III-mGluR in the mPFC. In this setting, III-mGluR activation reduces l-glutamate release. Perfusion with CLZ into the mPFC increased l-glutamate release in two phases in the mPFC (i.e., an initial rising phase and a late gradual attenuation phase). The late attenuation phase was abolished by perfusion with CPPG (III-mGluRs antagonist). To clarify the mechanisms of L-glutamate release in the mPFC caused by perfusion with CLZ into the mPFC, there needs to be a discussion as to whether GABAergic disinhibition in the mPFC is involved in CLZ-induced mPFC l-glutamate release. Local administration of a GABA_A_ receptor agonist, muscimol, into the mPFC has been shown to decrease monoamine release without affecting l-glutamate in the mPFC [17,18,19,49]. Taken together with previous demonstrations, therefore, the CLZ-induced l-glutamate release in the mPFC is probably independent on GABAergic transmission. 

### 4.3. Candidate Double-Edge Sword Mechanisms of the Action of CLZ

CLZ is known to protect against neuronal damage through the inhibition of several redox and proinflammatory responses [33,34], despite clinically high doses increasing the risk of several neuronal adverse effects [56]. Therefore, CLZ exhibits concentration-dependent actions that serve as a double-edged sword against cell protection. Enhanced astroglial release of L-glutamate and the endogenous NMDA-R agonist, D-serine, may be involved in the antipsychotic efficacy of CLZ [28]. It is established that NMDA-R agonism is central to the lower seizure threshold associated with CLZ therapy [25]. Survival analysis has suggested that lower limit of the therapeutic range of CLZ serum concentrations is 0.6 μM [55], whereas exceeding 4 μM significantly increases the risk of seizures [56]. Considering the diffusion rate through the dialysis probe, perfusion concentrations of 10 or 30 μM CLZ were considered therapeutic (this is estimated to be 0.96 and 2.8 μM in the brain tissue, respectively), whereas perfusion with 100 μM CLZ (9.6 μM in brain tissue) was considered toxic [55,56].

The concentration-dependent effects of CLZ on astroglial Sxc and hemichannel activities were determined in this study. We have already reported that Sxc activation in both the MDTN and mPFC inhibited MK801-induced L-glutamate release [13,14], and that the neuroprotective actions of memantine and amantadine were modulated by activating astroglial Sxc through the inhibition of neuronal NMDA-R [14,30]. Sxc is a rate-limiting factor in glutathione synthesis via its role in importing the extracellular L-cystine required for glutathione [30,59]. In turn, glutathione is capable of preventing neuronal damage induced by the redox response [30,59]. Additionally, glutathione inhibits the activities of ionotropic glutamate receptors, NMDA-R, and 2-amino-3-hydroxy-5-methyl- 4-isoxazolepropionate/glutamate receptors by binding to their glutamate recognition sites [60,61]. Unlike its neuroprotective action, Sxc activation in pathological conditions likely modulates neuronal damage via counter-exported L-glutamate. Indeed, oxidative stress is known to increase L-glutamate release from astroglial Sxc [59], and the inhibition of Sxc has been reported to attenuate cortical demyelination in an autoimmune encephalomyelitis model via the reduction of L-glutamate release from Sxc [62]. Based on these data, we determined the concentration-dependent effects of CLZ on Sxc activity using primary cultured astrocytes and mPFC l-glutamate release using microdialysis. However, we observed no effects of CLZ on Sxc activity. 

Gap-junctions and hemichannels are integral to neuronal excitability, synaptic plasticity, tripartite synaptic transmission, and homeostasis in the central nervous system. However, pathological conditions such as ischemia and excessive depolarization generate the persistent opening of gap-junctions and hemichannels, which leads to disruption of several homeostasis systems [36,37]. The dysfunction of glial gap-junctions contributes to attenuated information processing, causing cognitive impairment in schizophrenia [1]. Interestingly, an inhibitor of hemichannels and gap-junctions, CBX was reported to prevent hyperexcitability of the hippocampal neuronal network and persistent seizure discharge in a kainate-kindled epilepsy model. [37,38]. Therapeutically relevant concentrations of CLZ (lower than 3 μM) did not affect the astroglial l-glutamate release inhibited by CBX, whereas toxic concentrations of CLZ (higher than 10 μM) increased astroglial release. An in vivo microdialysis study also showed that l-glutamate release in the mPFC, as induced by perfusion with therapeutically relevant concentrations of CLZ (30 μM, with an estimated concentration in brain tissue of 2.8 μM) into the mPFC, was not affected by CBX, whereas the release induced by toxic concentrations of CLZ (100 μM, with an estimated concentration in brain tissue of 9.6 μM) was inhibited by CBX. These results suggest that toxic concentrations of CLZ weakly activate astroglial hemichannels and gap-junctions. 

The present demonstration that toxic concentrations of CLZ activated hemichannel activity can also be used to explain the mechanisms of CLZ-induced convulsion. Although this study could not detect the acute effect of therapeutically relevant concentrations of CLZ on l-glutamate release associated with hemichannels, we cannot deny that chronic administration of therapeutically relevant concentrations of CLZ weakly enhances astroglial hemichannels and gap-junctions. If CLZ weakly activates hemichannels and gap-junctions, this is a plausible mechanism explaining the advantage of CLZ over alternative antipsychotics for the management of treatment-refractory schizophrenia.

## 5. Conclusions

The present study not only demonstrated some mechanisms of pathophysiology of schizophrenia but also candidate mechanisms of the action of CLZ. First, the impairment of NMDA-R appears to enhance thalamocortical (MDTN–mPFC) l-glutamate release through thalamic (RTN–MDTN) GABAergic disinhibition. Second, therapeutically relevant concentrations of CLZ inhibit thalamocortical hyperglutamatergic transmission by activating presynaptic inhibitory III-mGluR in the mPFC. Third, the activation of astroglial hemichannels and gap-junctions by toxic concentrations of CLZ may contribute to CLZ-induced convulsion and other adverse effects.
